# On Filling, Teeth When the Pulp-Cavity Is Exposed, and Previously to the Death or Destruction of the Pulp

**Published:** 1849-07

**Authors:** Chapin A. Harris


### ARTICLE IV.

*On Filling Teeth when the Pulp-Cavity is Exposed, and previously to the Death or Destruction of the Pulp.* By CHAPIN A. HARRIS, M. D.

THE importance of the preservation of the vitality of the teeth is admitted by all having any knowledge of the laws of the animal economy. In proportion as it is diminished, does a tooth approach in its nature, a foreign substance, and hence is liable to become obnoxious to the living parts which surround it. But the deleterious influence resulting from the presence of a tooth after the destruction of the lining membrane, is not always occasioned by direct irritation produced by the organ itself; it is, perhaps, in the majority of cases, principally dependent upon an accumulation of morbid matter in the root of the tooth, and hence, by filling this, it may, in the majority of cases, be measurably prevented. Still, if the vitality of a tooth, after the lining membrane has become exposed, can be preserved, it is certainly better to do so, but with regard to the practicability of doing it, dentists, generally, are somewhat sceptical. They believe that inflammation and suppuration of the pulp, must follow, as a consequence, any operation which may be performed with a view to its accomplishment, and for a long time, the writer of this article entertained the same opinion. He has occasionally filled a tooth after the pulp had become exposed, for nearly fourteen years, but having regarded the operation more as experimental, than as one the success and utility of which were well established, he has refrained from recommending it very confidently to his professional brethren, until he should feel fully warranted in doing so. Even now, although he has performed the operation successfully in numerous instances, he feels considerable hesitancy with regard to the propriety of expressing his views upon the subject, nor would he at this time, had he not been frequently requested to do so.

Filling a tooth after caries has penetrated to the pulp-cavity, is an operation which has been frequently performed, but until recently it has been thought necessary to cap the pulp, previously to introducing the filling, so as to prevent the material employed in the operation from coming in contact with it. Dr. Koecker, formerly of Philadelphia, but now of London, was the first to propose this operation. The method of procedure he recommends, and which at the time of the publication of his principles of dental surgery, 1822, he says he had practiced for upwards of thirteen years, with success, is as follows:

First, after the removal of the caries, to give to the cavity a proper shape for the reception and retention of the filling; then to free the cavity by means of a little raw cotton, moistened with warm water, of any bone dust that may be in contact with the nerve.

Second, if the lining membrane is not wounded he proceeds at once, after perfectly drying the cavity, to fill it, which he does in the following manner: He places a small plate of very thin leaf lead upon the exposed nerve and the surrounding bone, and then proceeds to fill the cavity in the ordinary way with gold.

Third, when the lining membrane is wounded and bleeds, he cauterizes the part with an iron wire, heated to a red heat, using the precaution to prevent the wire from penetrating the exposed nerve. After the hemorrhage has been thus arrested, and an artificial cicatrix formed, the cavity is freed, in the manner as before described, from all loose extraneous matter, the nerve covered with sheet lead and a gold filling introduced.

For a more minute and detailed description of the method of procedure adopted by Dr. Koecker, the reader is referred to his Principles of Dental Surgery. In the hands of other practitioners the practice has been much less successful, and consequently, seems to have fallen into disrepute.

The reason which Dr. K. assigns for covering the nerve with lead, is, that he believes it to have a more "cooling and anti-inflammatory effect," than gold. That the practice may have been occasionally successful in his hands, the two cases which he adduces leave no room for doubt, but that the actual contact of any foreign substance with so exceedingly delicate and sensitive a tissue as the pulp of a tooth would, in a large majority of cases, be productive of irritation, there can be no question. Experience has fully established the correctness of this opinion.

Dr. Fitch recommends covering the nerve with a thin gold plate, previously to the introduction of the filling. This is certainly preferable to covering it with leaf lead, but unfortunately it is difficult in the majority of cases, to fit it with sufficient accuracy to the cavity of a tooth to prevent it from becoming displaced in the introduction of the filling. Besides, a cap of this sort would frequently occupy so much of the cavity, as to prevent the insertion of a filling in a sufficiently perfect manner to secure the preservation of the tooth.

The last named writer proposes another method of treating an exposed nerve, with a view to the preservation of its vitality, which he regards as preferable to either of the foregoing. It consists in the application of some powerful astringent to the exposed nerve, and keeping it in contact with it for a considerable length of time. Among the astringents which he has employed, are, alum, borax, and the gall-nuts, but he prefers the last. His method of using it is, to break the nut, take a small piece from the soft part, apply it to the exposed nerve, and fill the cavity with yellow wax. This may be replaced every ten or fifteen days until the sensibility of the nerve and lining membrane are so much reduced as to permit the introduction of a filling. This practice has not proved as successful as the remarks of Dr. F. upon the subject would lead one to expect. The result of the writer's experience will not by any means justify him in recommending its adoption. But were it otherwise, the great length of time required for the accomplishment of the object proposed to be gained by it, would, in most instances, constitute an insuperable objection to it.

In fact, so unsuccessful had every method of treatment proved, which had been proposed for the preservation of the vitality of a tooth after the pulp had become exposed, that the writer at one time had come to the conclusion that it was useless to attempt it. Whenever, therefore, he met with a molar or bicuspis in this condition, he advised its extraction; and his practice, with an incisor or cuspidatus, was to extirpate the nerve and fill the tooth. But in the spring of 1834, he was consulted by a female friend, about twenty-five years of age, in relation to her central incisores, which were in a decaying condition. Upon examination, the caries was found to be situated in the medial approximal surfaces, and had evidently penetrated the teeth to a considerable depth. With the exception of the discoloration occasioned by the caries of these two central incisores, the teeth of this lady were beautiful; they were pressed firmly against each other, but in other respects her teeth were well arranged. They were rather long, but of a medium size, of a light cream color, and neither very hard nor very soft.

The examination of her teeth having been completed, she requested that such operation might be performed as would be most likely to secure their preservation. Not being able to operate upon the teeth at that time, she was requested to call again. Punctual to the time appointed, she made her appearance. The teeth were separated with a file in the usual manner, and the decayed part, from first one, and then the other, removed; but in this part of the operation the lining membrane of each tooth was exposed and slightly wounded. The hemorrhage, however, was very slight, and soon subsided. Here was a dilemma from which the writer scarcely knew how to extricate himself. To extirpate the nerves, and thus rob the teeth of a great portion of their vitality, seemed, at this time, almost equivalent to their destruction. He was satisfied that the treatment proposed by Dr. Koecker could not be successfully adopted, and the size and shape of the cavities in the teeth would not admit of the application of gold caps, as recommended by Dr. Fitch. As a dernier resort, therefore, he determined to attempt to fill them over the exposed pulps, hoping that he might be able to introduce the fillings without coming in contact with these highly sensitive tissues. This, both to his own and his patient's great gratification, he succeeded in doing. Still, he was apprehensive that the operation would not prove permanently successful. He was fearful inflammation of the lining membrane would supervene, that the pulps would become tumefied, protrude through the openings, come in contact with the fillings, and that suppuration would ultimately take place. On the other hand, he hoped that by an operation of the economy like that which is set up when the crowns of the teeth are worn off, the pulp-cavities might be gradually filled up by a deposition of bony matter, or in other words, that the pulps might become wholly, or at least partially, ossified.

His fears were not immediately, nor his hopes ever fully, realized. He had frequent opportunities of examining the teeth for about four years after the operation had been performed, and the only inconvenience which seemed to result from it, was increased susceptibility to the action of heat and cold, but at the expiration of about six or eight weeks this subsided. The teeth retained that peculiar animated appearance which characterizes healthy living teeth, as he was informed, for nearly seven years, when they became slightly sore and painful to the touch. In a short time after, an abscess formed over each, and the teeth then assumed a brownish, muddy aspect.

That inflammation and suppuration of the pulps of these teeth should have occurred seven years after the operation was performed, may appear a little strange, and it would be interesting to know, if all the facts connected with the history of the case could be ascertained, whether the fillings had anything to do with the inflammation of the lining membrane which finally supervened, or whether it was dependent upon the state of the constitutional health or other causes? But the absence of such facts may render it improper to attempt a solution of these questions.

The apparent success, if the operation in the case just described be considered a failure, was certainly sufficient to justify its repetition; but regarding the preservation of the vitality of teeth in the condition in which these were, as almost impossible, the writer did not attempt to fill another, under similar circumstances, for about three years. The tooth in the next case was a bicuspis, and in a few weeks it became discolored, assuming an opaque and brownish appearance, indicating suppuration of the lining membrane and pulp. An alveolar abscess soon followed. Some two years after, he repeated the operation on two teeth, and in both cases with success, or at any rate the teeth retained their vitality and natural color for several months. From this time, he occasionally performed the operation, and with various success, but succeeding in by far the largest number of cases, until 1846. Still he regarded the operation in the light of experiment, and did not, from his own experience, feel safe in recommending it to others. He did, however, mention it to a number of professional friends, detailing the method of procedure which he adopted, and requesting that they would perform the operation whenever favorable cases should occur in their practice. Several of these gentlemen have performed the operation on a number of teeth, and on the majority, as he has been informed, with success. Dr. C. O. Cone has filled about fifty teeth after the lining membrane had become exposed, and up to the present time, so far as he has been able to ascertain, with success in forty-three or forty-four of the cases. He filled three teeth, a first left inferior molar, and the first superior bicuspides of a young lady, seventeen years of age, a relative of the writer, about eighteen months since. In two of the teeth, the lower molar and one of the bicuspides, the operation, up to this time, has proved successful; but in the other, inflammation and suppuration of the lining membrane and pulp took place about three months after the operation was performed, but the occurrence of alveolar abscess was prevented by the timely removal of the filling for the escape of the matter. After the diseased action which had been induced about the extremity of the root had subsided, the pulp-cavity, to the extremity of the root, was properly cleansed and filled.

Since the commencement of 1846, the writer has performed the operation on, probably, one hundred and fifty teeth, and up to this time, so far as he has been able to ascertain, on about twelve out of every thirteen with success. That inflammation and suppuration of the lining membrane and pulps of some of the teeth which have thus far escaped, may have taken place, he thinks highly probable, but if the vitality of four-fifths, taken under the most favorable circumstances, can be preserved, for from two to ten years, the operation will still be a most valuable one; and if it can be preserved for, from two to ten years, no good reason can be assigned why it should not be for a greater length of time. The loss of vitality, when it occurs a year or more after the operation has been performed, can scarcely be wholly attributed to the presence of the filling; it is doubtless in some degree, dependent upon constitutional or other causes.

The operation to be successful, must be carefully and skillfully performed, and only under certain circumstances; every particle of carious or decomposed bone, should be removed previously to the introduction of the gold, and this should be inserted in such a manner that it shall not come in contact with the exposed pulp; or, in other words, there should be a small space between the gold and pulp of the tooth. The introduction of the filling should be commenced on one side of the cavity, and fold after fold introduced, without carrying those immediately over or opposite the exposed pulp to the bottom of the cavity, until every part, except a small space next the nerve, is thoroughly filled, employing in the condensation of the metal only lateral pressure. When no more gold can be introduced, the extruding portion may be consolidated, and the surface finished in the manner as described in another place by the writer.* It is also important that the lining mem-

* See Principles and Practice of Dêntal Surgery.

brane be free from inflammation, and that the tooth never has, and that it does not ache at the time the operation is performed. Nor should the tooth be very soft, for in proportion as it is hard will be the prospect of success in the operation. The habit of body too, of the patient, should be as free from irritability as possible, and in other respects the general health should be good.

The writer will not enlarge further upon the operation in question; he considers his experience in it yet too limited to enable him to do so with profit. Although he is disposed to think favorably of it at present, its ultimate value, to some extent, remains to be determined. Hereafter, he may furnish the readers of the Journal with the result of his observations and experience upon the subject more fully.

